# Discovery of Novel Human Breast Cancer MicroRNAs from Deep Sequencing Data by Analysis of Pri-MicroRNA Secondary Structures

**DOI:** 10.1371/journal.pone.0016403

**Published:** 2011-02-08

**Authors:** Seongho Ryu, Natasha Joshi, Kevin McDonnell, Jongchan Woo, Hyejin Choi, Dingcheng Gao, William R. McCombie, Vivek Mittal

**Affiliations:** 1 Department of Cardiothoracic Surgery, Weill Cornell Medical College of Cornell University, New York, New York, United States of America; 2 Department of Cell and Developmental Biology, Weill Cornell Medical College of Cornell University, New York, New York, United States of America; 3 Laboratory of Plant Molecular Biology, Rockefeller University, New York, New York, United States of America; 4 Cold Spring Harbor Laboratory, New York, New York, United States of America; University of Edinburgh, United Kingdom

## Abstract

MicroRNAs (miRNAs) are key regulators of gene expression and contribute to a variety of biological processes. Abnormal miRNA expression has been reported in various diseases including pathophysiology of breast cancer, where they regulate protumorigenic processes including vascular invasiveness, estrogen receptor status, chemotherapy resistance, invasion and metastasis. The miRBase sequence database, a public repository for newly discovered miRNAs, has grown rapidly with approximately >10,000 entries to date. Despite this rapid growth, many miRNAs have not yet been validated, and several others are yet to be identified. A lack of a full complement of miRNAs has imposed limitations on recognizing their important roles in cancer, including breast cancer. Using deep sequencing technology, we have identified 189 candidate novel microRNAs in human breast cancer cell lines with diverse tumorigenic potential. We further show that analysis of 500-nucleotide pri-microRNA secondary structure constitutes a reliable method to predict bona fide miRNAs as judged by experimental validation. Candidate novel breast cancer miRNAs with stem lengths of greater than 30 bp resulted in the generation of precursor and mature sequences *in vivo*. On the other hand, candidates with stem length less than 30 bp were less efficient in producing mature miRNA. This approach may be used to predict which candidate novel miRNA would qualify as bona fide miRNAs from deep sequencing data with approximately 90% accuracy.

## Introduction

MicroRNAs (miRNAs) are small, non-coding RNAs (18∼23 nucleotide in size) that regulate gene expression by sequence specific binding to messenger RNA (mRNA), triggering either translation repression or RNA degradation [1]. miRNAs play important roles in various biological processes including cell growth, differentiation, and development [2], [3]. Abnormal miRNA expression has been reported in various diseases including cancer [4], [5], [6], [7], and are therefore considered to be promising diagnostic and therapeutic candidates for the treatment of human disease. miRNAs are transcribed as large primary microRNAs (pri-miRNAs, varying in length from a few hundred bases up to tens of kilobases) [8], which are further trimmed into precursor microRNAs (pre-miRNAs ∼75 nt) by the enzyme Drosha in the nucleus. The pre-miRNAs are exported to the cytoplasm where they are processed into mature miRNAs (∼22 nt) by the enzyme Dicer. Based on the thermodynamic stability of each end of this duplex, one of the strands is believed to be preferentially incorporated into the RNA-induced silencing complex (RISC), producing a biologically active miRNA and an inactive miRNA star sequence [9].

Currently, 1100 mature miRNAs have been discovered in mammalian systems and deposited in the publicly available miRNA database miRBase (Release 16; http://microrna.sanger.ac.uk/) [10], [11]. Although computational algorithms have predicted over 1500 human miRNAs [12], little over 1000 miRNAs (1100 in miRBase v16.0) have been assigned and validated in the human genome [11], [13]. Thus many miRNAs that occur in a cell have remained invalidated, and several others have not even been identified. A greater understanding of the roles of individual miRNAs requires comprehensive analysis of the full complement of such molecules and their relative abundance.

Recently, development of next generation sequencing technologies has revolutionized miRNA profiling in various model systems [14], [15], [16], [17]. Deep sequencing of miRNAs provides a highly quantitative estimate of known individual miRNA species, and has the potential for discovering novel miRNAs, even those that occur at low frequencies [18], [19]. Given that miRNAs contribute significantly to the pathophysiology of breast cancer by contributing to invasion and metastasis [20], [21], [22], [23], epithelial to mesenchymal transition [24], [25], and maintenance of breast stem cells [26], we have used a deep sequencing approach to identify novel miRNAs in human breast cancer cell lines that exhibit diverse tumorigenic potential. Multiple criteria, including frequencies of individual sequence reads, secondary structure of hairpins, thermodynamic stability, and the presence of star sequences were used to predict 189 novel miRNAs. We further developed a computational method based on the analysis of stem lengths in 500-nt pri-microRNA secondary structures to predict which candidate miRNA would qualify as bona fide miRNAs. Notably, accurate prediction was observed in 90% of the cases as judged by experimental validation.

## Results and Discussion

### Deep sequencing uncovers miRNAs in breast cancer cells

**Figure 1:**
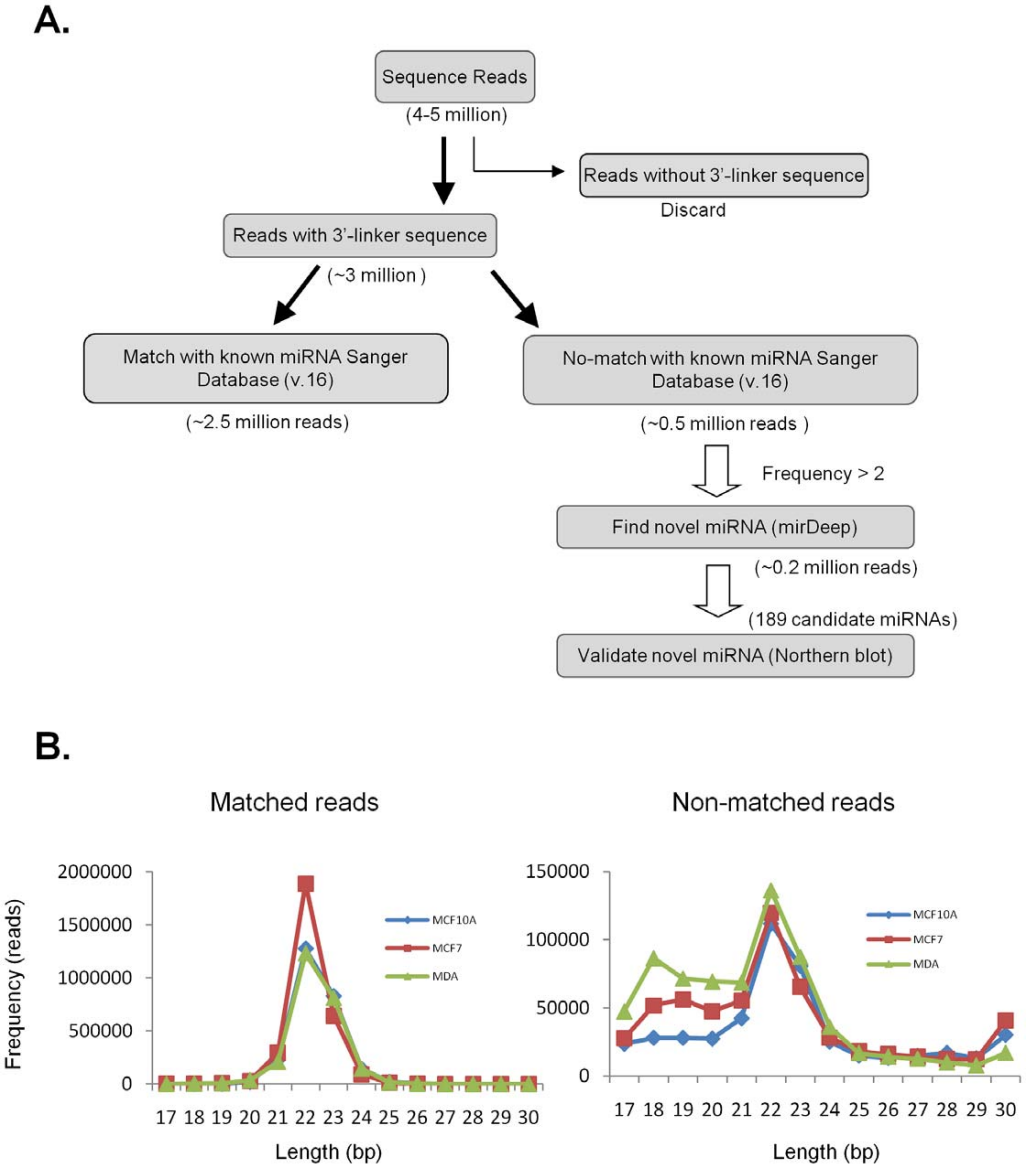
Analysis of deep sequencing data to identify novel miRNA. (A) Flowchart depicting analysis pipeline. Numbers of sequencing reads at each step of the analysis are shown in parentheses. (B) Size distribution (bp) shown for sequence reads that matched known miRNAs (matched reads) in miRBase and reads that did not match (non-matched reads) for human breast cell lines MCF10A, MCF7 and MDA-231 cells.

Total RNA isolated from human breast cell lines MCF10A (transformed primary breast epithelial cell, nontumorigenic), MCF7 (tumorigenic, nonmetastatic) and MDA-MB-231 (tumorigenic, metastatic) was size fractionated (15 to 32bp in length) and used to generate libraries for sequencing with the Illumina Solexa deep sequencing platform (Illumina 1× genome sequencer, Solexa). Approximately 4–5 million sequence reads obtained from each breast cancer cell line were subjected to quality control analysis to remove low quality sequences, and the remaining high confidence reads were used for identifying miRNAs as described in Methods. Next, the sequence reads were aligned to known miRNAs sequences in the miRNA database (miRBase v16) as shown in the schematic (**Fig. 1a**). Approximately 82% of the reads matched with the known human miRNAs, indicating that miRNAs had been successfully sequenced (**Supplementary Table S1**). These reads were eliminated from the data sets, and the remaining unmatched sequences (approx 0.5 million reads) were used for identifying novel miRNAs. Size distribution analysis showed that both the matched and unmatched categories predominantly consisted of reads approximately 22–23 nt in length (**Fig. 1b**). To identify novel miRNA, we considered sequence reads with a frequency ≥2, and approximately 0.2 million reads qualified this criteria.

### Identification of candidate novel miRNAs

To predict potentially novel miRNAs, we first combined all of the unmatched sequence reads from individual breast cancer cell lines and collapsed them to obtain a set of unique sequences. The reads were analyzed by miRDeep algorithm [18], which utilizes a probabilistic model of miRNA biogenesis to score compatibility of the position and frequency of sequenced RNA with the secondary structure of the miRNA precursor. Briefly, the reads were aligned to the genome database (human genome v18, UCSC genome browser) using the Megablast program [27]. Only sequences that aligned to the human genome were used for extracting ∼75bp potential pre-miRNA sequences. The ∼75bp pre-miRNA sequences were tested for their ability to form a characteristic hairpin structure using the Vienna RNA fold package v1.8 [28]. Sequences that formed reliable hairpin structures were further analyzed to determine features such as thermodynamics stability, presence of star sequences, and phylogenetic conservation [18]. Using a cut-off log-odds score of 1.0 (that is, results that are 10-fold more likely than random sequence to match the form of a predicted microRNA precursor, according to the miRDeep algorithm), we obtained a total of 189 potential novel miRNAs, which have the highest probability of being bona fide novel miRNAs (**Supplementary Table S2**). Of the 189 potential novel miRNAs, 30 candidates showed frequencies of >50 in both MCF10A, MCF7 or MDA-MB-231 breast cells (Table 1) and 27 candidate miRNAs contained the presence of star sequences. 7 candidate miRNAs had sequence homologies to known human miRNAs (1–2bp mismatch), and were located on different chromosome loci. 4 candidates matched with miRNAs of other species (1–2 bp mismatch), but did not match to human miRNAs. **Table 2** depicts the top 30 candidate miRNAs ranked on the basis of read frequencies.

**Table 1:** Characterization of 189 candidate miRNAs.

Category	Frequency
High confidence candidate miRNAs which has high frequency reads (>50)	30
Highly similar to known human miRNA (1∼2 mismatched) but located on different chromosome	7
No match with human miRNAs but highly similar to other species (0∼2 mismatched)	4
Presence of star sequence	27
Remaining	137

**Table 2:** A list of 30 high frequency novel candidate miRNAs in breast cancer cells.

NAME	SEQUENCE	length	MCF10A	MCF7	MDA	MIRBASE	STAR	LOCATION
**hsa-miR-B1**	GGCTGGTCCGAAGGTAGTGAGTTATCT	27	585	1347	1251	Novel	No	chr11:13304324–13304433[−]
**hsa-miR-B2**	CCTGCAGTAGCTGTTTCT	18	1	0	1651	Novel	No	chr9:73809165–73809274[−]
**hsa-miR-B3**	GGCTGGTCCGAGTGCAGTGGTGTTTACAACT	31	257	433	1007	Novel	No	chr13:98986564–98986673[−]
**hsa-miR-B4**	TAAAAGTAATTGTGGTATTTGC	22	0	0	57	1 mismatches with hsa-548d-5p	No	chr1: 81947441–81947550[+]
hsa-miR-B5	GAGCCCGGAGGGCGAGG	17	639	8	396	Novel	No	chr1:1172913–1173022[−]
**hsa-miR-B6**	AAGGTAGATAGAACAGGTCTTG	22	364	528	215	perfect match with mouse, dog, cow	Yes(22)	chr15:81221792–81221898[+]
**hsa-miR-B7**	CTGAGCAACATAGCGAGACCCCGTCTCTA	29	468	214	340	close to hsa-miR-1303 (chr5) in diff loc	No	chr16:3297692–3297801[−]
hsa-miR-B8	AAGCCATGTTACGAGCCTTAAGG	23	20	28	15	Novel	Yes(14)	chr6:133180097–133180198[+]
**hsa-miR-B9**	TGTGGTCTAGTGGTTAGGAT	20	169	111	157	close to cow (bta-mir-2476)	No	chrY:586300–586319[+]
**hsa-miR-B10**	GGGGGTGTAGCTCAGTGGTAGAGCA	25	209	46	92	1 mismatch with mmu-miR-1959	No	chr6:28834104–28834213[−]
hsa-miR-B11	GGCCAGCCACCAGGAGGGCTGC	22	1	15	14	Novel	Yes(5)	chr20:49502834–49502934[−]
hsa-miR-B12	TGTTGGTGTTTATGTTG	17	0	0	78	Novel	No	chr8:107080026–107080135[+]
hsa-miR-B13	CCGTGTTTCCCCCACGCTTT	20	49	29	206	Novel	No	chr17:8031210–8031319[+]
hsa-miR-B14	GTCTCCTTGTTATGGGGCAGTGCAG	25	153	10	29	Novel	No	chr1:153915582–153915691[−]
**hsa-miR-B15**	AAAGACATAGTTGCAAGATGGG	22	0	0	80	Novel	No	chr20:43767137–43767246[−]
hsa-miR-B16	GTGGGTGATGTTTGCTGACA	20	32	2	28	Novel	No	chr22:40334774–40334883[−]
hsa-miR-B17	GGGCTGTGATGTTTATTAGCTTCTGAGCTC	30	180	16	36	Novel	No	chr17:38359009–38359118[−]
hsa-miR-B18	GATGGTGATGATGCTGGTC	19	0	0	26	Novel		chr7: 91108780–91108889[+]
**hsa-miR-B19**	AAAAGGGGGCTGAGGTGGAG	20	8	0	133	Novel	No	chr11:121527997–121528099[−]
hsa-miR-B20	CCAAGGAAGGCAGCAGG	17	19	22	125	Novel	No	chr1:202811178–202811287[−]
**hsa-miR-B21**	TATGTGTGTGTGCTTGTATAT	21	1	0	88	close to mouse (mmu-miR-669)	No	chr8:129122276–129122385[+]
hsa-miR-B22	TCCCCAGCACCTCCACCA	18	114	1	11	Novel	No	chr7:73108306–73108415[−]
**hsa-miR-B23**	TGAGGAATATGGTGATC	17	0	0	48	Novel	No	chr1:33878065–33878174[+]
hsa-miR-B24	GTTCTTGTAGTTGAAATACAACGATG	26	29	12	51	Novel	No	chr5:105917028–105917137[+]
hsa-miR-B25	TTGGCCATGGGGCTGCGCGG	20	33	42	36	Novel	No	chr19:764562–764671[+]
hsa-miR-B26	ATCCCACCACTGCCACCA	18	59	21	3	one mismatch with hsa-miR-1260	No	chr11:95714237–95714346[+]
**hsa-miR-B27**	CCAGGAATCCTGCTGTGGTGGA	22	8	0	50	Novel	Yes(3)	chr11:121532093–121532199[−]
**hsa-miR-B28**	TGTCCTTGCTGTTTGGAGATAA	22	68	21	8	close to cow (bta-mir-2355)	Yes(14)	chr2:207682944–207683054[−]
hsa-miR-B29	ATGTGGGCTAGTTTCAGACAGGT	23	7	11	42	Novel	No	chr1:28778841–28778945[−]
hsa-miR-B30	CACCTTGCGCTACTCAGGTCTGC	23	13	47	15	Novel	No	chr22:29457597–29457619[+]

Candidate miRNAs in bold were used to evaluate miRNA expression using Northern blotting.

### Experimental validation of candidate novel miRNAs

**Figure 2:**
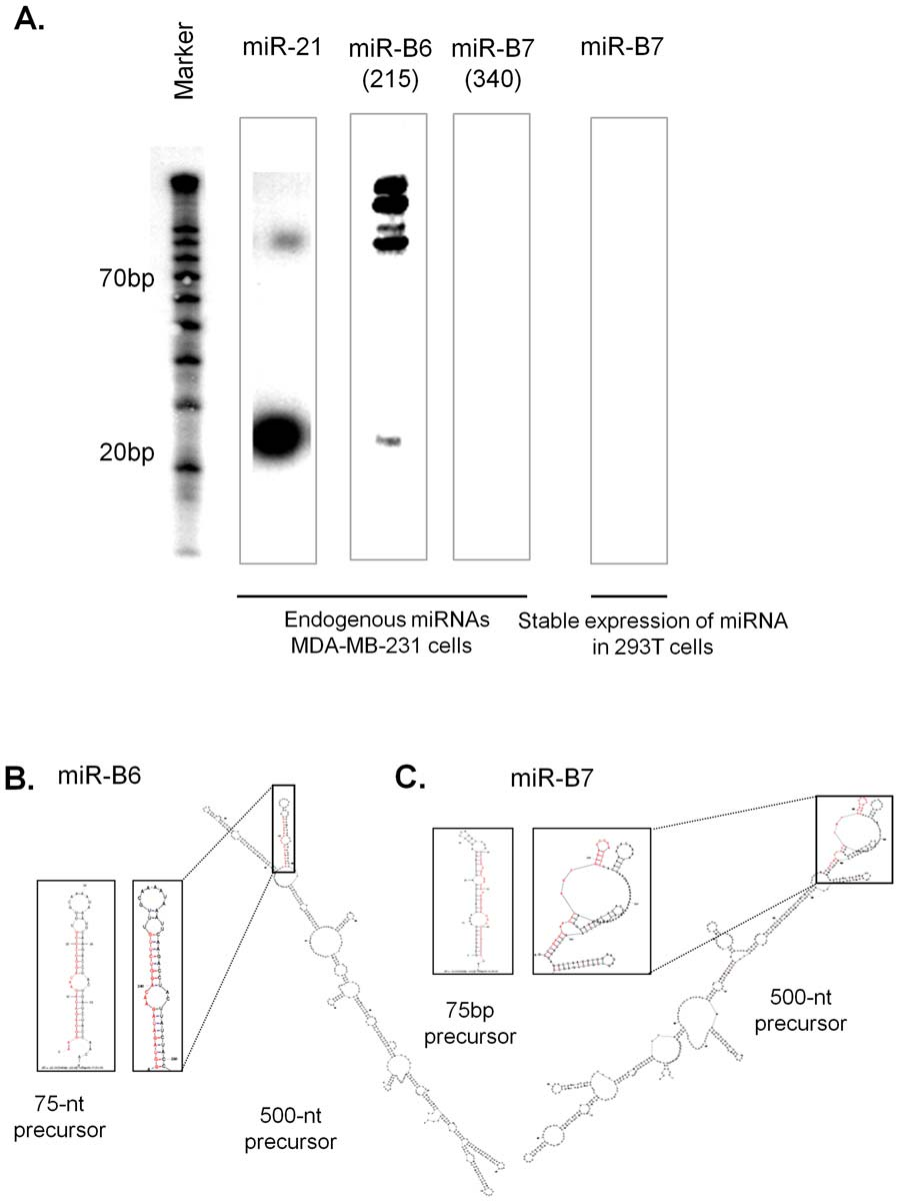
Validation of candidate miRNAs by northern blot analysis and secondary structure prediction of precursor sequences. (A) 20µg of RNA (enriched for small RNA fraction) was isolated from MDA-MB-231 cells and fractionated on a 7% polyacrylamide gel and hybridized to anti-sense oligo probes corresponding to miRNAs indicated above to detect processed 70-nt precursor and a 22-nt mature miRNA. In some cases, 500nt- precursors were cloned in lentiviral vectors to generate 293T cells stably expressing precursor miRNAs. RNA harvested from the transfected cells was used for northern blot analysis. A highly abundant known miRNA, hsa-miR-21 was used as a control. Decade marker was used as a size marker for small RNAs. (B) Secondary structures were predicted by RNA fold program using either a ∼75-nt precursor sequence or the 500-nt precursor sequence. The stem and loop structure of pre-miRNA are boxed. The location of mature miRNA sequence is denoted in red.

To determine which of these predicted candidates were bona fide miRNAs, a subset of the novel miRNA candidates from top 30 candidate miRNAs (**Table 2**) were subjected to experimental validation. It is important to note that, in principle, a miRNA is considered validated when expression of its ∼75-nt precursor and ∼22-nt mature processed fragment can be demonstrated [29]. In this context, two candidate miRNAs miR-B6 and miR-B7 depicted in **Table 2** with comparable frequencies and high similarity with known miRNAs were first selected for northern blot analysis of total RNA. hsa-miR-21, a highly abundant known miRNA in our deep sequencing data was used as a control. Surprisingly, we observed that only miR-B6 showed both the precursor and mature processed fragment in northern blot analysis (**Fig. 2A**), even though both candidate miRNAs had qualified miRDeep parameters including presence of typical fold-back hairpin structures. There is a distinct possibility that the suboptimal sensitivity of northern blotting may be the cause for our failure to detect endogenous mature miRNAs. To discount this possibility, we cloned the 500bp of miR-B7 precursor sequence (with GFP) in a lentiviral vector and generated 293T cells stably expressing the miR-B7 precursor. Again, overexpression of mir-B7 did not reveal either the pre-miRNA or the mature mir-B7 product consistent with previous results (**Fig. 2A**).

**Figure 3:**
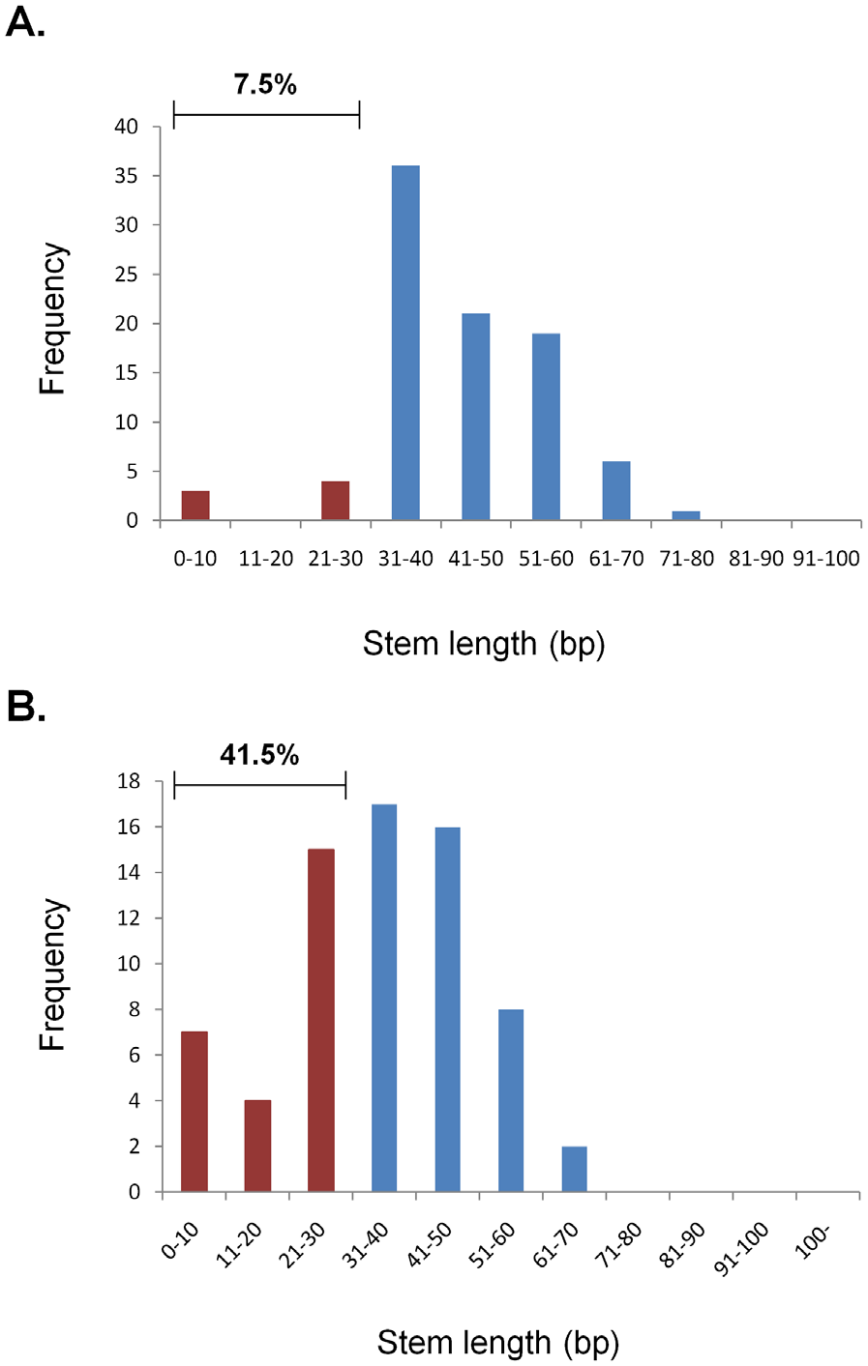
Distribution of the length of stem in known and unknown miRNA sequence reads. The pri-miRNA secondary structures in both known miRNAs and candidate miRNAs were predicted by RNAfold program. The secondary structures of pre-miRNA were located and extracted to measure the length of stem in both known miRNAs (A) and candidate miRNAs (B). Blue and red bars represent the distribution of longer stem length (cutoff >30) and short stem length, respectively. Read frequencies with stem length of >30 or <30 are indicated.

**Figure 4:**
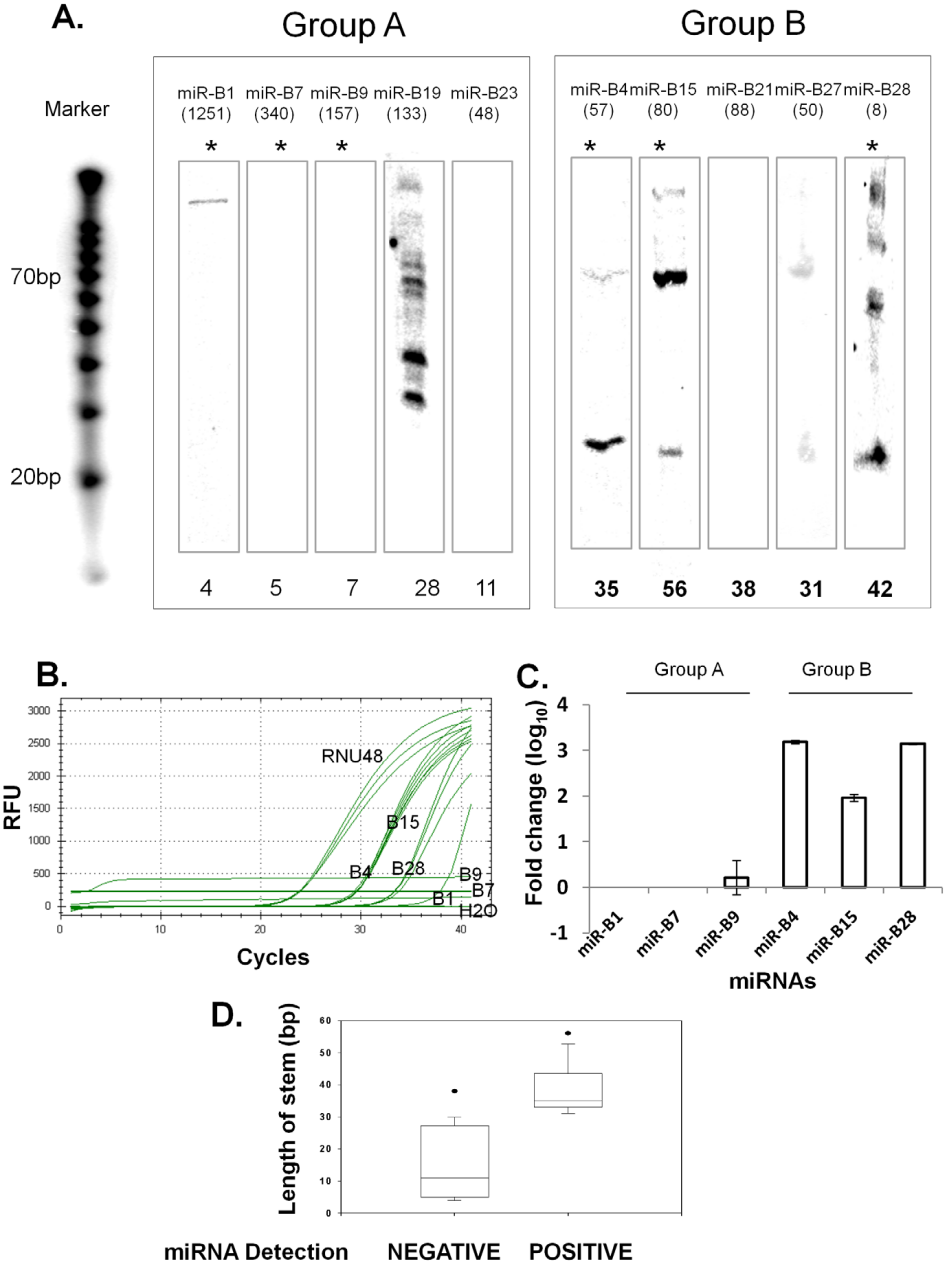
Northern blot and Taqman QPCR analysis showing that novel candidate miRNAs with stem lengths of ≥30bp may comprise *bona fide* miRNAs. (A) The expression of miRNA was examined by northern blotting using end-labeled oligonucleotide probes. A total of 10 candidate miRNAs were selected from Table 2 and their 500-nt precursors cloned in plasmids and overexpressed in 293T cells to detect processed 70-nt pre-miRNA and a mature miRNA. The numbers in parentheses represent the read frequency in MDA-MB-231 cells obtained from deep sequencing. The numbers at the bottom indicate the length of stem in individual candidate miRNAs. A synthetic ^32^P-labeled RNA marker was used as a size marker. Asterisk indicate miRNAs selected for QPCR validation. Taqman miRNA assay showed expression of candidate miRNAs from each group by Ct values (B) and fold change (C). RNU48 was used as positive control and no-template control (NTC) used as negative control. (D) Box plot graph showing correlation between validated miRNA and non-validated miRNA with their stem length. Mann-Whitney test showing that the two groups are statistically different (p<0.001).

The stem-loop hairpin structure is a valuable, but not discriminative, characteristic of pre-miRNAs, because previous studies have shown that folding free energy and structural criteria often used to generate miRNA precursors are not the most informative when it comes to distinguishing precursors from other non-miRNA conserved hairpins [30]. Therefore, to determine the possible cause for the failure of miR-B7 to generate a mature sequence, we examined its RNA secondary structure by generating fold-back hairpin structure derived from a standard ∼75-nt precursor sequence or from a larger ∼500-nt precursor as depicted for known miRNA miR-21 (**Supplementary Fig. S1**). Notably, while the ∼75-nt precursor sequence generated a characteristic miRNA stem-loop structure, the 500-nt precursor sequence yielded a distorted stem-loop structure (**Fig. 2C**), as determined by the reduction in the length of the stable stem. In contrast, both the ∼75-nt and the ∼500-nt precursor for miR-B6 generated a robust stem-loop structure with an intact stem (**Fig. 2B**). This observation led to the hypothesis that the analysis of the stem-loop structure obtained from a 500-nt precursor may be an important determinant for evaluating whether a mature miRNA will be generated *in vivo* from precursor sequences. To test this hypothesis, we generated a positive sample dataset by selecting the 100 most highly expressed miRNAs, including miR-21, from our deep sequencing data that matched known miRNAs in the miRBase. These reads were aligned to the genome database (human genome v18, UCSC genome browser) using the Megablast program [27]. Next, for each miRNA, we extracted ∼500-nt pri-miRNA sequences, generated secondary structures and measured the length of stem containing mature miRNA sequence (length between hairpin loop junction and bulge defined as more than four consecutive non-paring nucleotides or starting point of other branch of stems). The results showed that a majority (>90%) of highly expressed known miRNAs in our positive sample dataset consisted of stem lengths more than 30bp in length and only 7.5% had stem lengths of less than 30 bp (**Fig. 3A**). Using this information from the training set, we performed identical analysis on 189-candidate novel miRNAs identified by miRDeep (top 30 candidate miRNAs listed in **Supplementary Fig. S2**). Interestingly, the frequency of the length of stem showed a typical normal distribution pattern with stem lengths ranging from 0–70bp and 41.5% constituting stem lengths less than 30 bp (**Fig. 3B**). Based on the data from the training set, we inferred that novel candidate miRNAs with stem lengths of ≥30bp may comprise bona fide miRNAs. To test this hypothesis, we selected the top 20 most highly expressed candidate miRNAs with sequence homologies with other species and performed northern blot analysis on either total RNAs harvested from breast cancer cells or RNA extracted following expression of precursors cloned in plasmids to detect processed 70-nt precursor and a mature 22-nt miRNA. The results summarized in **Supplementary Table S3** shows that candidate miRNAs with longer stem lengths (cutoff ≥30, Group B in **Fig. 4**) resulted in the generation of precursor and mature sequences (4 out of 5). On the other hand, candidates with short stem length (cutoff <30, Group A in **Fig. 4**) did not produce mature miRNA (0 out of 6). These results suggest that our approach can be used to predict which candidate miRNA would qualify as *bona fide* miRNAs from miRNA deep sequencing with about 90% accuracy. Although northern blot analysis provides a direct evidence to determine precursor and mature form of miRNA, there is a possiblity that the sensitivity of notthern blotting may not be sufficient enough to detect lowly expressed miRNAs. Therefore, we selected three miRNAs each from two groups, designed custom Taqman miRNA probes (Applied Biosystems) and performed miRNA QPCR assay. As expected, consistent with the northern blot data, three candidate miRNAs from Group B, miR-B4, miR-B15, miR-B28 were detected, but none of three candidate miRNAs from Group A were detected by QPCR assay (Fig. 4B and C). Notably, comparison of validated candidate novel miRNAs and their stem lengths between group A and B by Mann-Whitney statistical test showed that two groups are significantly different (p<0.01) (Fig. 4D).

Given that the folding free energy analysis of pre-miRNA secondary has proven insufficient to discriminate miRNA precursors from other structures, additional criteria have been considered, including preference for a 5′ Uracil [31], stem or bulge symmetry [31] [32] , and frequencies of 3-nt elements combining base- pairing and sequence characters [33], lower number and reduced size of bulges and internal loops [30]. Although these parameters have improved miRNA prediction, this prediction has not been tested in experimental settings. In this study, we have demonstrated that analysis of stem lengths generated by 500-nt pri-miRNA secondary structure can be used to identify bona fide novel miRNAs from deep sequencing data. Perhaps experimental evaluation of a larger number of candidate miRNAs will be necessary to accurately determine the robustness of this method in the future.

## Materials and Methods

### Cell lines and expression of miRNA

Human breast cell lines MCF10A (nontumorigenic), MCF7 (tumorigenic, nonmetastatic) and MDA-MB-231 (tumorigenic, metastatic) were obtained from ATCC. MCF10A cells were grown in DMEM/F12 (Mediatech) with EGF (Peprotech), Hydrocortizone (Sigma), Cholera Toxin (Sigma) and Insulin (Sigma) as instructed in ATCC guideline. MCF7 and MDA-MB-231 cells were grown in DMEM (Mediatech) with 10% FBS (Hyclone), 5% glutamine (Mediatech), and 5% antibiotics-antimycotic solution (Mediatech). To express miRNA in cells, ∼500bp of pri-miRNA were amplified and cloned into pZEO lentiviral construct in fusion with a GFP reporter (gift from Patrick Paddison, Cold Spring Harbor Lab). Lentivirus was generated by co-transfection of pZEO constructs with packaging constructs pMD2G and psPAX2 into 293T cells as described [34]. Culture medium containing lentivirus was collected at 48 and 72 h after transfection. The titer of lentivirus was determined by infecting 293T cells and evaluating % GFP+ cells. Cells were infected with pzeo-empty or with pzeo-pri-miRNA at multiplicity of infection (MOI) of ∼10. GFP signal was used to sort cells stably expressing precursor miRNA by flow cytometry.

### Total RNA extraction, library construction and high-throughput sequencing

Total RNA was extracted from breast cancer cells using TRIZOL as per the manufacturer's instructions (Invitrogen). Approximately, 20µg of total RNA was fractionated through a 15% polyacrylamide-Urea gel (Sequagel, National diagnostic) along with ^32^-P labeled 19–24bp oligonucleotide-delimiting markers. Small RNA fraction was extracted from the gel slice corresponding to the delimiting markers and used for generating libraries for deep sequencing. Briefly, small RNAs were ligated with Modban (Linker 1 from IDT). Ligated samples were separated again on 15% Acrylamide/Urea gel. The gel fragments corresponding to ∼36–41 nt were excised. Small RNA was purified from the gel fragments, and 5′ sequencing linker was added using T4 RNA ligase and ligated product (∼68–73 nt) separated on a 15% Polycrylamide/Urea gel. This RNA was amplified using Superscript III Reverse transcriptase (Invitrogen) and PCR amplification (Taq polymerase, Roche). Amplified cDNA were separated on 2% Low melt agarose gel (SeaKem) and the integrity of the small RNA libraries tested on 2100 Agilent Bioanalyzer (Agilent Technologies, Santa Clara, CA). Each library was sequenced using Solexa 1× genome sequencer (Illumina, San Diego, CA).

### Data Analysis

Sequencing images from Solexa 1× genome sequencer were analyzed using the Illumina Pipeline v1.3.2 software to remove background noise and to extract the first 36 bases of the runs. Sequence reads were aligned to the hg18 genome (UCSC genome browser) using the Eland software (Illumina, San Diego, CA). Next the linker sequences were identified and trimmed from individual reads using a customized Perl script. Reads in which the linker sequences were either mutated or absent were discarded. Next, the high confidence trimmed reads were aligned to known miRNAs available in the miRBase (v16, www.mirbase.org), to obtain sequences that either matched or did not match to known miRNAs and their frequencies calculated. Unmatched sequences were collapsed to obtain a set of unique reads. The reads that passed were analyzed by miRDeep algorithm to predict novel miRNAs [18], [19]. Briefly, reads were mapped to human genome (human genome v18, UCSC genome browser) using Megablast [27] to identify reads with perfect matches. Reads that aligned to more than five positions in the genome were removed from the dataset, as these may comprise of repetitive sequences. The remaining reads were subjected to noncoding RNA database (NONCODE, v2.0, http://www.noncode.org) to remove the contamination of human noncoding RNA such as snRNA, snoRNA, r-RNA, t-RNA [35]. From the rest potential precursors (∼75bp) were excised to generate secondary stem-loop structure using Vienna RNAfold package v1.8 (http://rna.tbi.univie.ac.at/) [28]. Finally, miRDeep assigned a score to each read based on: (a) seed sequence homology to known human miRNA, (b) presence of a star sequence, (c) minimum free energy (RNAfold), (d) energetic stability (Randfold), (e) frequency of reads that correspond to Dicer processing. A final score was assigned and the minimum total score by default was 1.

To measure the length of stem in the secondary structure of miRNAs, ∼500-nt precursor sequences were extracted and secondary structure generated using Vienna RNAfold package. The stem length was measured by counting the nucleotides between hairpin loop junction and the base of the stem/bulge defined as more than four consecutive non-paring nucleotides or starting point of other stem branches.

### Northern blotting

Small RNAs were enriched using mirVana small RNA extraction kit as per the manufacturer's manual (Ambion, Applied Biosystems, CA). 20 µg of enriched RNA was fractionated through a 7% polyacrylamide gel with decade RNA makers (Ambion, Applied Biosystems) for an hour and transferred into Hybond-N+ positively charged nylon transfer membrane (Amersham) using semidry membrane transfer apparatus (Biorad) for 45min at 100mA. Following transfer, RNA was fixed to the membrane using a UV-crosslinker. Oligo probes obtained from Sigma were labeled using gamma-^32^P ATP (>3000 Ci/mmol) and T4 polynucleotide kinase (10U/ul, NEB) for an hour at 37°C. Unincorporated isotope was removed using a quick spin column (Roche) as per the manufacturer's instructions. Membranes were hybridized with labeled probes using Ultrahyb-Oligo solution (Ambion, Applied Biosystems) overnight at 42°C. After hybridization, membranes were washed twice in 2X SSC (Ambion, Applied Biosystems) and exposed to film.

### Taqman miRNA QPCR

Taqman probes for miR-B1, miR-B4, miR-B7, miR-B9, miR-15 and miR-28 were designed using Custom TaqMan Small RNA Assays (Applied Biosystems, CA). Human small nucleolar RNA, RNU48 was used as endogenous controls (Applied Biosystems). 10ng of RNA were used to make cDNA using Taqman microRNA assays kit and QPCR was performed as per the manufacturer's manual (Applied Biosystems, CA).

## Supporting Information

Figure S1 **Pre- and pri-miRNA sequence of hsa-miR-21 and its secondary structure.** (A) The sequence in bold represents 75-nt pre-miRNA and mature miRNA sequences depicted in red. (B) A predicted stem-loop secondary structure of 75-nt pre-miR-21. Sequences corresponding to the mature miRNAs are shown in red. (C) A predicted stem-loop secondary structure derived from a 500-nt pri-miR-21. The length of the stem is measured by counting nucleotides from the stem-loop junction to the end of stem.
(TIF)

Figure S2 **Comparison of the secondary structures of 75-nt pre-miRNA and 500-nt pri-miRNA for highly expressed 30 candidate miRNAs.** Nucleotide sequences represent the primary sequence of precursor miRNA. Parentheses, “(” and “)” indicate the base pairing. Dots represent unpaired nucleotide. miRNAs in blue were validated by northern blotting. “Pass” and “Fail” indicate pass or fail to form loop-stem structure, respectively.
(DOC)

Table S1 Statistical values in each step of the pipeline to find candidate miRNAs in various breast cancer cells.
(DOC)

Table S2 A list of candidate miRNAs predicted by mirDeep algorithm.
(DOC)

Table S3 A lists of miRNA evaluated by Northern blot.
(DOC)
