# Supplementary figures and images for: Discovery of Novel Human Breast Cancer MicroRNAs from Deep Sequencing Data by Analysis of Pri-MicroRNA Secondary Structures
Source: PLoS One. 2011 Feb 8;6(2):e16403. doi: 10.1371/journal.pone.0016403 (PMC3035615; doi:10.1371/journal.pone.0016403)

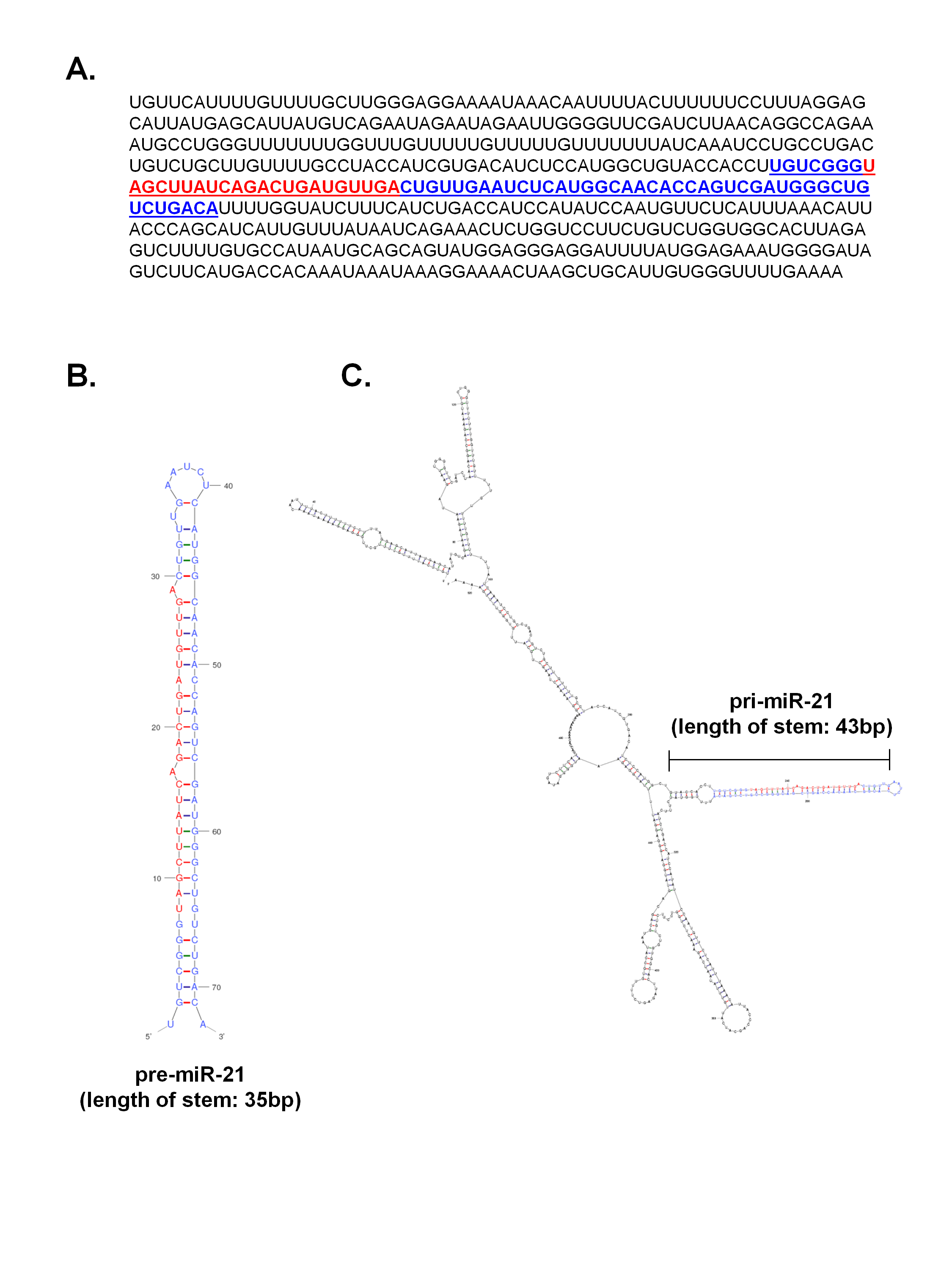

Supplement: Figure S1 — Pre- and pri-miRNA sequence of hsa-miR-21 and its secondary structure. (A) The sequence in bold represents 75-nt pre-miRNA and mature miRNA sequences depicted in red. (B) A predicted stem-loop secondary structure of 75-nt pre-miR-21. Sequences corresponding to the mature miRNAs are shown in red. (C) A predicted stem-loop secondary structure derived from a 500-nt pri-miR-21. The length of the stem is measured by counting nucleotides from the stem-loop junction to the end of stem. (TIF) [file pone.0016403.s001.tif]
