# Supplementary material for: Discovery of Novel Human Breast Cancer MicroRNAs from Deep Sequencing Data by Analysis of Pri-MicroRNA Secondary Structures
Source: PLoS One. 2011 Feb 8;6(2):e16403. doi: 10.1371/journal.pone.0016403 (PMC3035615; doi:10.1371/journal.pone.0016403)
Supplement: Figure S2 — Comparison of the secondary structures of 75-nt pre-miRNA and 500-nt pri-miRNA for highly expressed 30 candidate miRNAs. Nucleotide sequences represent the primary sequence of precursor miRNA. Parentheses, “(” and “)” indicate the base pairing. Dots represent unpaired nucleotide. miRNAs in blue were validated by northern blotting. “Pass” and “Fail” indicate pass or fail to form loop-stem structure, respectively. (DOC) [file pone.0016403.s002.doc]

**Figure S2.** Comparison of ~75-nt pre-miRNA and the same sequence from pri-miRNA secondary structure for 30 candidate miRNAs

miR-B1

GGCUGGUCCGAAGGUAGUGAGUUAUCUCCAUUGAUAGUUCAGUCUGUAACAGAUCAAACUCCUU

(..(((((.(..((.(.(((..((((......))))..))).)))....).)))))..)..... (-12.70)

).)))))))))).(((((((((((((.((((((.((.((((((((((((((((.(((((((..(

--> fail

miR-B2

CCUGCAGUAGCUGUUUCUCUACUUACAGCCACAGCUACUGCUGGGCA

((.(((((((((((..((........))..))))))))))).))... (-21.90)

((((((((((((((..((........))..))))))))))).)))).

--> pass

miR-B3

GGCUGGUCCGAGUGCAGUGGUGUUUACAACUAAUUGAUCACAACCAGGUACAGCACUGCUUCAUUUGACUAGCCUU

((((((((.(((((.((..((((((((......(((....)))....))).)))))..)).))))))))))))).. (-29.50)

((((((((.(((((.((..((((((((......(((....)))....))).)))))..)).)))))))))))))).

--> pass

miR-B4

UAAAAGUAAUUGUGGUAUUUGCCAUUAAAGUACUGCAAAAGCCGAAAUUACUUUUGCA

(((((((((((.((((.(((((............))))).)))).))))))))))).. (-17.40)

(((((((((((.((((.(((((............))))).)))).))))))))))).)

--> pass and confirmed with northern blot

miR-B5

GUGCUCUCUGGAGAUGCCAGUCAGUGCCUGAGAGCCCGGAGGGCGAGG

.((((((((((.(...((((.......))).)..)))))))))))... (-18.40)

)))))).((((.....)))).)))))))...))))))))).))).(((

--> fail

miR-B6

AAGGUAGAUAGAACAGGUCUUGUUUGCAAAAUAAAUUCAAGACCUACUUAUCUACCAACA

..(((((((((...((((((((..(.........)..))))))))..))))))))).... (-22.60)

..(((((((((...((((((((..(.........)..))))))))..)))))))))..))

--> pass and confirmed with northern blot

miR-B7

GAGGUGGGAGGAUCACUUGAGCCAGGAGUUUCAGACCAGCCUGAGCAACAUAGCGAGACCCCGUCUCUA

(((..(((.(..((.((((.((((((.(((...)))...)))).))..)).)).))..))))..))).. (-23.00)

(((((.(..((.(((((..((((..(((((((.......(((((((..(((...(((((...)))))..

--> fail

miR-B8

AAGCCAUGUUACGAGCCUUAAGGACAUUGAAGUCGUUAAGGUCCCUGAGAAUGGCUAUA

.((((((.(((.(..((((((.(((......))).))))))..).)))..))))))... (-23.60)

.((((((.(((.(..((((((.(((......))).))))))..).)))..))))))...

--> pass

miR-B9

CCCAUACAGGAUCUGCAAAAAGCACUCUGCCCUGUGGUCUAGUGGUUAGGAU

.((.(((.(((((.(((....((.....))..)))))))).)))....)).. ( -9.40)

((......)))))))))....)))))))))....))).))))).)..)))))

--> fail

miR-B10

GGGGGUGUAGCUCAGUGGUAGAGCACAUGCUUUGCAUGUGUGAGGCCCCGGGUUCGAUCCCCGGCACCU

((((((..(((((.(.(((...((((((((...))))))))...)))).)))))..))))))....... (-32.30)

)...))).)))....)))))).((((((((...))))))))((((..(((((.......)))))..)))

--> fail

miR-B11

GGCCAGCCACCAGGAGGGCUGCGUGCCACCCGGGCAGCUCUGCUGCUCACUGGCAGU

.(((((....(((.((((((((.((.....)).)))))))).)))....)))))... (-29.70)

.(((((....(((.((((((((.((.....)).)))))))).)))....)))))..)

--> pass

miR-B12

UGUUGGUGUUUAUGUUGUUUCUAUCAUUCAGGUGCUAUGAUGAGAGGGGAUUAAGAGGGGCUAUGAGCAGCGACAGU

(((((.((((((((..((..((...((((.....(......).....))))....))..)))))))))).))))).. (-19.90)

((.(((..............))).)))))))))(((.....(((((((((.......(((((....((.....((((

--> fail

miR-B13

AGCGCCUGUCUAGUAAACAGGAGAUCCUGGGUUCGAAUCCCAGCGGUGCCUCCGUGUUUCCCCCACGCUUU

((((..((....(.(((((((((((((((((.......))))).)))..)))).))))).)..)))))).. (-19.90)

((((..((....(.(((((((((((((((((.......))))).)))..)))).))))).)..))))))))

--> pass

miR-B14

GUCUCCUUGUUAUGGGGCAGUGCAGCUGUAGGCCAAGCUGUAUCUGUUUGGGAAGGGAGAAAA

.(((((((....(.(((((((((((((........))))))).)))))).)..)))))))... (-26.70)

.(((((((....(.(((((((((((((........))))))).)))))).)..)))))))...

--> pass

miR-B15

AAAGACAUAGUUGCAAGAUGGGAUUAGAAACCAUAUGUCUCAUCAGCACCCUAUGUCCUUUCU

...(((((((.(((..((((((((............)))))))).)))..)))))))...... (-19.40)

((((((((((.(((..((((((((............)))))))).)))..)))))).))))))

--> pass and confirmed with northern blot

miR-B16

UUAGCAAUCAGUAAUCUGCCAUGUCGCCGUGUGAAGUGAUAAGCAGGAGUGAGUGGGUGAUGUUUGCUGACA

(((((((.((...(((..((((....((.(((..(....)..))))).))).)..)))..)).))))))).. (-20.40)

...((.....))...)))).)).)))).)))))))))))).....((((((((..((.....))..))..))

--> fail

miR-B17

GUUGAGCUAGAUCUAUGUUAUUGCUCUCUCUGGGGCUGUGAUGUUUAUUAGCUUCUGAGCUC

((((((((((....(((((((.(((((....))))).)))))))...)))))))...))).. (-16.10)

))..)))))).)))))..........))))...))).))))))))))).((((...((((((

--> fail

miR-B18

GAUGGUGAUGAUGCUGGUCUAUUCUAGAUCCUUGUCCGCCAAAUAUGGUCUAUGAGCCAGUAGCAUCAGCAAUCCC

..((.(((((.(((((((....((((((((..(((.......))).)))))).))))))))).))))).))..... (-23.20)

((((.(((((.(((((((....((((((((..(((.......))).)))))).))))))))).))))).).)))))

--> pass

miR-B19

UCCCCUUAGUUUCCUUUAAGAGUGAUAAAAAUGGAAAAGGGGGCUGAGGUGGAG

((((((((((..(((((....((.......))...)))))..))))))).))). (-22.40)

((.(((((((..(((((....((.......))...)))))..))))))).))))

--> pass

miR-B20

CCAAGGAAGGCAGCAGGUUCCAGGCCAUGCUUAGUCCCCUAGUGUC

...(((..((((((((((.....))).))))..))).)))...... (-11.00)

((.(((..((((((((((.....))).))))..))).))).).)))

--> pass

miR-B21

GUAUUGUUUACGUGCAUGUGUUUGUGUGUAUAUGUUUGUGCAUGUGUGCAUAUGUGUGUGUGCUUGUAUAU

((((.((.((((..(((((((.((..((((((....))))))..)).)))))))..)))).))..)))).. (-25.00)

((((.((.((((..(((((((.((..((((((....))))))..)).)))))))..)))).))..))))))

--> pass

miR-B22

UCCCCAGCACCUCCACCAUGAAGCCUUUAAAAAAAAUGUGGGGCCGGGUGCAGUGGCU

...(((((((((((.((((..................))))))..))))))..))).. (-15.77)

..))))))..)))))).......)))))........))))))))))))).)...(((.

--> fail

miR-B23

UUAUGCUCAUGUUCAGUUUAUAGAUGAGGAAUAUGGUGAUC

....((.(((((((.(((....)))...))))))))).... ( -7.80)

)........)))..))..)))))))))))...)))))....

--> fail

miR-B24

GUUCUUGUAGUUGAAAUACAACGAUGGUUUUUCAUAUCACUGGUCGUGGUUGUAGUCCAUGCGAGAAUAA

(((((((((...((..((((((.(((..(............)..))).)))))).))..))))))))).. (-19.20)

(((((((((...((..((((((.(((..(............)..))).)))))).))..))))))))).(

--> pass

miR-B25

GGGCAGCGUGUGGCUGAAGGUCACCAUGUUCUCCUUGGCCAUGGGGCUGCGCGG

(.(((((..((((((.((((............))))))))))...))))).).. (-22.00)

(.(((((..((((((.((((............))))))))))...))))).)))

--> pass

miR-B26

AUCCCACCACUGCCACCAUUAUUGCUACUGUUCAGCAGGUGCUGCUGGUGGUGAUGGUGAUAGUCUGGUGGGGGCG

.(((((((((((.(((((((((..(((.....((((....)))).)))..))))))))).))))..)))))))... (-37.80)

.(((((((((((.(((((((((..(((.....((((....)))).)))..))))))))).))))..))))))))))

--> pass

miR-B27

CCAGGAAUCCUGCUGUGGUGGAGAGGCAUAGACUCAUGAGUCCACCACAAUCGGAUACUUGCU

.((((.((((...((((((((((((.......))).....)))))))))...)))).)))).. (-24.70)

(.(((.((((...((((((((((((.......))).....)))))))))...)))).))))))

--> pass

miR-B28

AUCCCCAGAUACAAUGGACAAUAUGCUAUUAUAAUCGUAUGGCAUUGUCCUUGCUGUUUGGAGAUAA

(((.((((((((((.(((((..(((((((.........))))))))))))))).))))))).))).. (-24.20)

(((.((((((((((.(((((..(((((((.........))))))))))))))).))))))).))).)

--> pass and confirmed with northern blot

miR-B29

AAUAGUGAAGCUGGCCUAAAUGUUGUAAUCUGGUAUAUGGCAUGUGGGCUAGUUUCAGACAGGU

.....(((((((((((((.((((..((........))..)))).)))))))))))))....... (-26.50)

.....(((((((((((((.((((..((........))..)))).))))))))))))).....((

--> pass

miR-B30

GAAUCUGAGAAGGCGCACAAGGUUUGUGUCCAAUACAGUCCACACCUUGCGCUACUCAGGUCUGC

..(((((((..((((((..((((..(((..(......)..))))))))))))).))))))).... (-23.60)

(.(((((((..((((((..((((..(((..(......)..))))))))))))).))))))).).)

--> pass
