# Supplementary material for: Discovery of Novel Human Breast Cancer MicroRNAs from Deep Sequencing Data by Analysis of Pri-MicroRNA Secondary Structures
Source: PLoS One. 2011 Feb 8;6(2):e16403. doi: 10.1371/journal.pone.0016403 (PMC3035615; doi:10.1371/journal.pone.0016403)
Supplement: Table S1 — Statistical values in each step of the pipeline to find candidate miRNAs in various breast cancer cells. (DOC) [file pone.0016403.s003.doc]

**Table S1.** Statistical values in each step of the pipeline to find candidate miRNAs in various breast cancer cells

|  | **MCF10A** | **MCF7** | **MDA-MB-231** |
| --- | --- | --- | --- |
| **Flow cell Reads** | **6,509,611** | **6,680,794** | **7,739,594** |
| **Reliable Reads (Illumina QC)** | **4,363,442**  **(67%)** | **4,616,346**  **(69%)** | **4,656,589**  **(60.2%)** |
| **Reads with linker sequence** | **3,056,709**  **(70.4%)** | **3,565,093**  **(77.4%)** | **3,197,724**  **(68.8%)** |
| **Reads that match with known human miRNA**  **(Sanger DB, V.16)** | **2,536,616**  **(82.9%)** | **2,973,587**  **(83.4%)** | **2,465,790**  **(77.1%)** |
| **No match** | **520,093** | **591,506** | **731,934** |
| **Unique reads in no match pool** | **97,353** | **166,020** | **145,068** |
| **Predicted novel miRNA by mirDeep**  **(189 unique reads)** | **138** | **125** | **155** |
