# Supplementary material for: Discovery of Novel Human Breast Cancer MicroRNAs from Deep Sequencing Data by Analysis of Pri-MicroRNA Secondary Structures
Source: PLoS One. 2011 Feb 8;6(2):e16403. doi: 10.1371/journal.pone.0016403 (PMC3035615; doi:10.1371/journal.pone.0016403)
Supplement: Table S2 — A list of candidate miRNAs predicted by mirDeep algorithm. (DOC) [file pone.0016403.s004.doc]

**Table S2.** A list of candidate miRNAs predicted by mirDeep algorithm.

| **NAME** | **SEQUENCE** | **MCF10A** | **MCF7** | **MDA_B** | **MIRBASE** | **STAR** | **LOCATION** |
| --- | --- | --- | --- | --- | --- | --- | --- |
| **hsa-miR-B1** | **GGCTGGTCCGAAGGTAGTGAGTTATCT** | **585** | **1347** | **1251** | **Novel** | **No** | **chr11:13304324-13304433[-]** |
| **hsa-miR-B2** | **CCTGCAGTAGCTGTTTCT** | **1** | **0** | **1651** | **Novel** | **No** | **chr9:73809165-73809274[-]** |
| **hsa-miR-B3** | **GGCTGGTCCGAGTGCAGTGGTGTTTACAACT** | **257** | **433** | **1007** | **Novel** | **No** | **chr13:98986564 -98986673[-]** |
| **hsa-miR-B4** | **TAAAAGTAATTGTGGTATTTGC** | **0** | **0** | **57** | **1 mismatches with hsa-548d-5p in diff loc** | **No** | **chr1: 81947441 -81947550[+]** |
| **hsa-miR-B5** | **GAGCCCGGAGGGCGAGG** | **639** | **8** | **396** | **Novel** | **No** | **chr1:1172913-1173022[-]** |
| **hsa-miR-B6** | **AAGGTAGATAGAACAGGTCTTG** | **364** | **528** | **215** | **perfect match with mouse, dog, cow** | **Yes(22)** | **chr15:81221792 -81221898[+]** |
| **hsa-miR-B7** | **CTGAGCAACATAGCGAGACCCCGTCTCTA** | **468** | **214** | **340** | **close to hsa-miR-1303 (chr5) in diff loc** | **No** | **chr16:3297692 -3297801[-]** |
| **hsa-miR-B8** | **AAGCCATGTTACGAGCCTTAAGG** | **20** | **51** | **15** | **Novel** | **Yes(14)** | **chr6:133180097-133180198[+]** |
| **hsa-miR-B9** | **TGTGGTCTAGTGGTTAGGAT** | **169** | **111** | **157** | **close to cow (bta-mir-2476)** | **No** | **chrY:586300-586319[+]** |
| **hsa-miR-B10** | **GGGGGTGTAGCTCAGTGGTAGAGCA** | **209** | **46** | **92** | **1 mismatch with mouse (mmu-miR-1959)** | **No** | **chr6:28834104-28834213[-]** |
| **has-miR-B11** | **GGCCAGCCACCAGGAGGGCTGC** | **42** | **57** | **83** | **Novel** | **No** | **chr20:20265597:20265576[-]** |
| **hsa-miR-B12** | **TGTTGGTGTTTATGTTG** | **0** | **0** | **78** | **Novel** | **No** | **chr8:107080026-107080135[+]** |
| **hsa-miR-B13** | **CCGTGTTTCCCCCACGCTTT** | **49** | **29** | **206** | **Novel** | **No** | **chr17:8031210 -8031319[+]** |
| **hsa-miR-B14** | **GTCTCCTTGTTATGGGGCAGTGCAG** | **153** | **10** | **29** | **Novel** | **No** | **chr1:153915582-153915691[-]** |
| **hsa-miR-B15** | **AAAGACATAGTTGCAAGATGGG** | **0** | **0** | **80** | **Novel** | **No** | **chr20:43767137 -43767246[-]** |
| **hsa-miR-B16** | **GTGGGTGATGTTTGCTGACA** | **55** | **2** | **28** | **Novel** | **No** | **chr22:40334774-40334883[-]** |
| **hsa-miR-B17** | **GGGCTGTGATGTTTATTAGCTTCTGAGCTC** | **180** | **16** | **36** | **Novel** | **No** | **chr17:38359009 -38359118[-]** |
| **hsa-miR-B18** | **GATGGTGATGATGCTGGTC** | **0** | **0** | **51** | **Novel** | **No** | **chr7: 91108780-91108889[+]** |
| **hsa-miR-B19** | **AAAAGGGGGCTGAGGTGGAG** | **8** | **0** | **133** | **Novel** | **No** | **chr11:121527997-121528099[-]** |
| **hsa-miR-B20** | **CCAAGGAAGGCAGCAGG** | **19** | **22** | **125** | **Novel** | **No** | **chr1:202811178-202811287[-]** |
| **hsa-miR-B21** | **TATGTGTGTGTGCTTGTATAT** | **1** | **0** | **88** | **close to mouse (mmu-miR-669)** | **No** | **chr8:129122276-129122385[+]** |
| **hsa-miR-B22** | **TCCCCAGCACCTCCACCA** | **114** | **1** | **11** | **Novel** | **No** | **chr7:73108306 -73108415[-]** |
| **hsa-miR-B23** | **TGAGGAATATGGTGATC** | **0** | **0** | **48** | **Novel** | **No** | **chr1:33878065 -33878174[+]** |
| **hsa-miR-B24** | **GTTCTTGTAGTTGAAATACAACGATG** | **29** | **12** | **51** | **Novel** | **No** | **chr5:105917028-105917137[+]** |
| **hsa-miR-B25** | **TTGGCCATGGGGCTGCGCGG** | **33** | **42** | **36** | **Novel** | **No** | **chr19:764562-764671[+]** |
| **hsa-miR-B26** | **ATCCCACCACTGCCACCA** | **59** | **21** | **3** | **one mismatch with hsa-miR-1260 (chr14) in diff loc** | **No** | **chr11:95714237 -95714346[+]** |
| **hsa-miR-B27** | **CCAGGAATCCTGCTGTGGTGGA** | **8** | **0** | **50** | **Novel** | **Yes(3)** | **chr11:121532093-121532199[-]** |
| **hsa-miR-B28** | **TGTCCTTGCTGTTTGGAGATAA** | **68** | **21** | **8** | **close to cow (bta-mir-2355)** | **Yes(14)** | **chr2:207682944-207683054[-]** |
| **hsa-miR-B29** | **ATGTGGGCTAGTTTCAGACAGGT** | **7** | **11** | **42** | **Novel** | **No** | **chr1:28778841-28778945[-]** |
| **hsa-miR-B30** | **CACCTTGCGCTACTCAGGTCTGC** | **13** | **47** | **15** | **Novel** | **No** | **chr22:29457597-29457619[+]** |
| has-miR-B31 | ATCCAGCGGTTGTCAGCTATCCAGGCTC | 24 | 0 | 21 | Novel | No | chr5:138642368-138642395[+] |
| has-miR-B32 | TGCCATGATGAGAATTTATCTGAGG | 16 | 6 | 6 | Novel | No | chr11:69173901-69173925[+] |
| has-miR-B33 | GGTTGGCAAGGTCAGTGTGCCTTT | 33 | 4 | 10 | Novel | No | chr6:28801789-28801898[+] |
| has-miR-B34 | TGAAGCGCCTGTGCTCTGCCGAG | 22 | 10 | 12 | Novel | No | chr15:83724874-83724896[+] |
| has-miR-B35 | TCCTGGCTGGCTCGCCC | 20 | 13 | 21 | Novel | No | chr22:20595055-20595071[+] |
| has-miR-B36 | TATGTGTATGTGTATGTGTGTAA | 15 | 11 | 3 | Novel | No | chr4:36136782-36136804[+] |
| has-miR-B37 | GAGAGAGATGAGAGCCTTTTAGGCTGAGG | 17 | 21 | 10 | Novel | No | chr1:45016079 -45016191[+] |
| has-miR-B38 | TAACTGTGATGCCAGAATCTGAGTT | 7 | 0 | 17 | Novel | No | chr1: 54531091-54531200[-] |
| has-miR-B39 | CGCCGCTTTCTGGGCTCGCT | 16 | 1 | 14 | Novel | No | chr5:72745611-72745630[+] |
| has-miR-B40 | ATGTCATAGCAGGTGTACC | 0 | 49 | 0 | Novel | No | chr11:86220115-86220133[+] |
| has-miR-B41 | TTGTGAAGAAAGAAATTCTT | 16 | 8 | 4 | Novel | No | chr10:35408520-35408629[-] |
| has-miR-B42 | AAAGGTAATTGCAGTTTTTCCC | 16 | 17 | 4 | Novel | No | chr6:99679221-99679242[+] |
| has-miR-B43 | TGTGAGAGTATGTTCGCATCT | 8 | 0 | 14 | Novel | No | chr20:59563268-59563288[+] |
| has-miR-B44 | CTAGATGATGAAGAAAGAGGCTCTGACC | 3 | 8 | 8 | Novel | No | chr17:2972262-2972289[+] |
| has-miR-B45 | GAGGTGGGATCCCGAGGCC | 8 | 3 | 25 | Novel | No | chr16:46096231-46096249[+] |
| has-miR-B46 | GGGTGCGGGCCGGCGGGGT | 22 | 9 | 5 | Novel | No | chr6:13757440-13757458[+] |
| has-miR-B47 | GGCCAGCCACCAGGAGGGCTGC | 1 | 15 | 14 | Novel | **Yes(5)** | chr20:49502834-49502934[-] |
| has-miR-B48 | TGCAGGAACTTGTGAGTCT | 8 | 0 | 0 | Novel | No | chr22:16755883-16755901[+] |
| has-miR-B49 | CTCCAATGATGCCAGGCTGAGGATG | 13 | 2 | 19 | Novel | No | chr16:22506791-22506815[+] |
| has-miR-B50 | AGGAGCTACGTTTTTTGGT | 2 | 38 | 0 | Novel | No | chr4:79376056-79376074[+] |
| has-miR-B51 | AGTGTTATGGGCCCGGAGCG | 16 | 1 | 12 | Novel | No | chr4:185815723-185815742[+] |
| has-miR-B52 | GTGCACATTGTTAGAGCTTGGAGTT | 9 | 2 | 7 | Novel | No | chr3:39427550-39427574[+] |
| has-miR-B53 | CAGCCCGGATCCCAGCCCACTT | 7 | 3 | 20 | Novel | **Yes(6)** | chr19:6367421-6367522[-] |
| has-miR-B54 | GCTTGTTGTGATTCCTCCATTTT | 32 | 0 | 0 | Novel | No | chr5:180461528-180461550[+] |
| has-miR-B55 | TTTTGCTGCAGTGTGGATGGTA | 4 | 1 | 1 | Novel | No | chr12:103918546-103918567[-] |
| has-miR-B56 | AGCGCGGGCTGAGCGCTGCCAGTC | 12 | 6 | 7 | Novel | **Yes(26)** | chr5: 92982149-92982255[-] |
| has-miR-B57 | AGGGGCGCGGCCCAGGAGCTCAGA | 7 | 2 | 13 | Novel | No | chr11:9189208-9189231[+] |
| has-miR-B58 | CTCCCTGTCCTCCAGGAGCTCA | 12 | 6 | 11 | **hsa(shift-339-5p)** | **Yes(9)** | chr7:1029094-1029198[-] |
| has-miR-B59 | AGCCCGCCCCAGCCGAGGTTCT | 14 | 4 | 8 | Novel | **Yes(9)** | chr14:22495984-22496091[-] |
| has-miR-B60 | TGGGTGATGCAGTGAGATGCTGT | 7 | 0 | 9 | Novel | **Yes(9)** | chr3:47505232-47505335[-] |
| has-miR-B61 | AGCTGGTGCTGTGAATCAGG | 3 | 0 | 22 | Novel | No | chr3:168737436-168737455[+] |
| has-miR-B62 | TCCCTGTCCTCCAGGAGCTT | 11 | 3 | 11 | Novel | No | chr2:64596870-64596889[+] |
| has-miR-B63 | TGGACTGAGGGAAATAATCTATTCTGAGGCT | 19 | 2 | 4 | Novel | **Yes(6)** | chr17:72069293-72069401[+] |
| has-miR-B64 | GAGAGCAGTGTGTGTTGCCTGGG | 9 | 2 | 15 | Novel | No | chr9:96612080-96612102[+] |
| has-miR-B65 | AAAAGGCGGGAGAAGCCCCA | 3 | 0 | 25 | Novel | No | chr10:127498362-127498381[+] |
| has-miR-B66 | TTAATTTTTTGTTTCGGTCACT | 2 | 3 | 11 | Novel | No | chr2:208327785-208327806[+] |
| has-miR-B67 | CAGGGCTGGCAGTGACATGGGT | 0 | 28 | 0 | Novel | No | chr3:114796455-114796476[+] |
| has-miR-B68 | TGCCTGAGGGAGTAAGAGTCC | 7 | 0 | 2 | Novel | No | chr7:129134478-129134498[-] |
| has-miR-B69 | AATAGCTCAGAATGTCAGTTCTGT | 5 | 2 | 15 | Novel | No | chr8:44490399-44490422[+] |
| has-miR-B70 | AAGGTGAATTAGTTGGA | 0 | 29 | 0 | Novel | No | chr5:167565100-167565116[+] |
| has-miR-B71 | TGTCCTCTAGGGCCTGCAGTCT | 5 | 2 | 10 | Novel | No | chr22:34061703-34061724[+] |
| has-miR-B72 | ACTGGCCTGGGACTACCGGGGGT | 11 | 11 | 2 | Novel | No | chr16:533337-533359[+] |
| has-miR-B73 | ATATGGGTTTACTAGTTGGT | 7 | 2 | 1 | Novel | No | chr1:23243390-23243409[+] |
| has-miR-B74 | GTCCCGGTCGCCGCGGTTCGCCG | 10 | 6 | 6 | Novel | No | chr16:33873565-33873587[+] |
| has-miR-B75 | TCCGTCCGCGTGTGGAAGGTC | 0 | 0 | 14 | Novel | No | chr22:49209672-49209692[+] |
| has-miR-B76 | TGAGGGGAGAATGAGGTGGAGA | 7 | 3 | 10 | Novel | No | chr1:203646753-203646774[+] |
| has-miR-B77 | TCTGTGAGACCAAAGAACTACT | 3 | 6 | 2 | Novel | Yes | chr1:241576091-241576193[+] |
| has-miR-B78 | ACAGTGAGGTAGAGGGAGTGC | 5 | 0 | 9 | Novel | No | chr4:181583702-181583722[+] |
| has-miR-B79 | GCCTGTGAGTGGTGGGTTTGTG | 7 | 1 | 5 | Novel | No | chr15:36390312-36390333[+] |
| has-miR-B80 | TGGAAAGGATGAAGAGCTGACTGATGGGG | 5 | 6 | 2 | Novel | No | chr17:7750337-7750365[+] |
| has-miR-B81 | TGAGGATATGGCAGGGAAGG | 4 | 6 | 5 | Novel | No | chr2:134601171-134601190[+] |
| has-miR-B82 | AGTCCTCTGGGTGGTTATGAGC | 0 | 0 | 9 | Novel | **Yes(5)** | chr2:239020714-239020808[+] |
| has-miR-B83 | TCAGGGCCGAAGGGTGGAAGC | 0 | 11 | 4 | Novel | No | chr9:134917207-134917227[+] |
| has-miR-B84 | GAGGCTGATGTGAGTAGACCACTT | 18 | 4 | 0 | Novel | No | chr18:44349083-44349106[+] |
| has-miR-B85 | TCCGGTAGGGTTCGGGCCTTCC | 2 | 6 | 11 | Novel | No | chr11:15981752-15981773[+] |
| has-miR-B86 | CAAAGGGTGAGAATACCAGCCA | 0 | 0 | 5 | Novel | No | chr10:130711466-130711487[+] |
| has-miR-B87 | ATATGTAAGTATATGTGTGTG | 7 | 12 | 0 | Novel | No | chr9:135476606-135476626[+] |
| has-miR-B88 | TGTCTTTCCTTGTTGGAGCAGG | 0 | 11 | 2 | Novel | No | chr10:134697081-134697102[+] |
| has-miR-B89 | TTCAGCCAGGCTAGTGCAGTCT | 5 | 8 | 4 | Novel | No | chr10:37560601-37560622[+] |
| has-miR-B90 | TCAGGTGTGGAAACTGAGGCAGG | 7 | 4 | 4 | Novel | **Yes(5)** | chr6:33773885-33773989[+] |
| has-miR-B91 | CAGCTGTGATGAAGTGCCCATCTGAGC | 8 | 1 | 11 | Novel | No | chr16:86832253-86832279[+] |
| has-miR-B92 | AAGGAGCTGGAGAAGCT | 2 | 18 | 0 | Novel | No | chr1:245372682-245372698[+] |
| has-miR-B93 | TGAGGCCGAGAAGGCAACCGCGA | 7 | 0 | 9 | Novel | No | chr1:148473740-148473762[+] |
| has-miR-B94 | TAGTGAGTTAGAGATGCAGAGCC | 8 | 1 | 4 | Novel | No | chr15:88351000-88351022[+] |
| has-miR-B95 | TCAGCAGGCAGGCTGGTGCAGCCC | 5 | 3 | 4 | Novel | No | chr22:44865603-44865626[+] |
| has-miR-B96 | TCCAACGTGGATACACCCGGGAGG | 8 | 2 | 5 | Novel | No | chr1:28706464-28706487[+] |
| has-miR-B97 | GACCTCCTGGGATCGCATCTGGAGAGTGCCT | 5 | 1 | 10 | Novel | No | chr3:186644879-186644909[+] |
| has-miR-B98 | AGATGTCCAGCCACAATTCTCG | 4 | 8 | 4 | Novel | No | chr9p;130194734-130194755[+] |
| has-miR-B99 | TGGCTGGCCCGTGGGGCGAG | 0 | 3 | 2 | Novel | No | chr2:215803232-215803251[+] |
| has-miR-B100 | CTGTCCTAAGGTTGTTGAGTT | 4 | 12 | 0 | **mmu ; 2-3 mismatches** | **Yes(4)** | chrX:69159417-69159518[+] |
| has-miR-B101 | AAAAGCTGTCCACTGTAGAGTT | 0 | 0 | 11 | Novel | No | chr9:107826885-107826906[+] |
| has-miR-B102 | TGTCACTCTTGTGTGTTGCAGA | 1 | 0 | 10 | Novel | No | chr17:16294579-16294600[+] |
| has-miR-B103 | TCGGGCCCCAGATGGCCGGCGGC | 7 | 0 | 8 | Novel | No | chr7:9114346-9114368[+] |
| has-miR-B104 | TACCCCAGGATGCCAGCATAGTT | 9 | 0 | 6 | Novel | No | chr5:138639889-138639911[+] |
| has-miR-B105 | ACGCCTCTTCAGCGCTGTCTT | 7 | 0 | 6 | Novel | No | chr1:148966283-148966303[-] |
| has-miR-B106 | TGCAGCTCTGGTGGAAAATGGAGA | 5 | 1 | 4 | Novel | No | chr8:8943373-8943396[+] |
| has-miR-B107 | TCTTGTAGCAGGTATTTTCCCT | 0 | 1 | 8 | Novel | No | chr1:129050743-129050764[+] |
| has-miR-B108 | AAGATTGTTGCTTCTTTCC | 0 | 0 | 6 | Novel | No | chr10:64572977-64572995[+] |
| has-miR-B109 | TGCAATGATGGGTAAGCTGAGG | 5 | 0 | 2 | Novel | No | chr7:157366328-157366349[+] |
| has-miR-B110 | GCCCAGGATGAAACTCTGACA | 2 | 2 | 7 | Novel | No | chr4:1707066-1707086[+] |
| has-miR-B111 | CCACGATGATGTCTGCTGAGTA | 10 | 2 | 3 | Novel | No | chr16:4537136-4537157[+] |
| has-miR-B112 | CCGGTCCCAGGAGAACCTGCAGA | 5 | 1 | 7 | Novel | No | chr9:4396984-43970069[+] |
| has-miR-B113 | TTGGGCATATGTGTATATATGT | 4 | 1 | 1 | Novel | No | chr20:12269307-12269328[+] |
| has-miR-B114 | CTCCTGGGGCCCGCACTCTCGC | 4 | 6 | 3 | Novel | **Yes(4)** | chr11:34919952-34920054[+] |
| has-miR-B115 | CCGGCCACGCGCCTCTGTCTTG | 4 | 4 | 5 | Novel | No | chr7:746806-746827[+] |
| has-miR-B116 | CTTCCATCTCCATCACCTTGAGC | 0 | 6 | 4 | Novel | **Yes(10)** | chr16:80124989-80125092[+] |
| has-miR-B117 | GCACTGTGATGAGAAGGACTCTGAGG | 1 | 11 | 1 | Novel | No | chr9:136480608-136480633[+] |
| has-miR-B118 | TGTGTTGCATGTGTGTATATGT | 0 | 15 | 0 | Novel | No | chr3:168323558-168323579[+] |
| has-miR-B119 | TCGCCGGGGAGCCAAGCACCGCT | 0 | 0 | 8 | Novel | No | chr20:59262353-59262375[+] |
| has-miR-B120 | AGGCCGCGGAGCCAGCGACGAC | 2 | 3 | 4 | Novel | No | chrx:129339318-129339339[+] |
| has-miR-B121 | AAGCCCAGAAGCTGGAAACTGCAGT | 3 | 4 | 2 | Novel | No | chr2:69256179-69256203[+] |
| has-miR-B122 | ATAGTCACAAGTAAGAGAGTGA | 1 | 2 | 1 | Novel | No | chr14:26294424-2629444[+] |
| has-miR-B123 | CAAAAGTAACTGTGGTTTTGCA | 0 | 6 | 8 | Novel | No | chr1:182019105-182019126[+] |
| has-miR-B124 | CGGGAGCGGCGGGGGCGG | 5 | 1 | 4 | Novel | No | chrx:135863157-135863174[+] |
| has-miR-B125 | GAATTATAACTCGGTGCTCTGT | 6 | 0 | 8 | Novel | No | chr3:191383262-191383283[+] |
| has-miR-B126 | TTATCATTGTTTTAGTGTTTGT | 6 | 1 | 3 | Novel | No | chr9:37147027-37147048[+] |
| has-miR-B127 | AACTAGCTCTGTGGATCCTGA | 1 | 1 | 11 | Novel | No | chr8:92286898-92286918[+] |
| has-miR-B128 | ATCAGGGCTTGTGGAATGGGAAG | 2 | 9 | 1 | Novel | No | chr2:96827752-96827774[+] |
| has-miR-B129 | CTTGCTCTGCTCTCCCTTGTACT | 5 | 2 | 5 | Novel | No | chr14:11298667-11298689[+] |
| has-miR-B130 | CTCCGGTGATGACCACAGGGACTGAGA | 2 | 0 | 3 | Novel | No | chr20:35448756-35448782[+] |
| has-miR-B131 | CCAGCCTACTGGAGGATAAGAG | 1 | 6 | 2 | Novel | No | chr8:46799699-46799720[+] |
| has-miR-B132 | AATGAGAAAATGTCCCACATTT | 4 | 0 | 0 | Novel | No | chr1:34509681-34509702[+] |
| has-miR-B133 | TCTGCAGCCCTGGAGCCCCCTGT | 5 | 0 | 5 | Novel | No | chr20:30707811-30707833[+] |
| has-miR-B134 | TGTGTGTGTATAAATATGTATATA | 1 | 2 | 0 | Novel | No | chr10:35535197-35535220[+] |
| has-miR-B135 | TGGGCTAAGGGAGATGATTGGGTA | 1 | 6 | 3 | Novel | **Yes(4)** | chrx:153650065-153650088[+] |
| has-miR-B136 | AAGTAGTTTCATGATAAAGGGT | 1 | 0 | 3 | Novel | No | chr1:9950037-9950058[+] |
| has-miR-B137 | GGGCCATGATGGTGGCTGAGCCTG | 5 | 1 | 1 | Novel | No | chr11:47305975-47305998[+] |
| has-miR-B138 | AGTGGATGATGCACTCTGTGCA | 0 | 0 | 7 | Novel | No | chr2:12256716-12256737[+] |
| has-miR-B139 | CTGCAGTGATGAGCGAGCCCGGCCTGAGCTG | 5 | 1 | 0 | Novel | No | chr7:100589823-100589853[+] |
| has-miR-B140 | AATGGTGAGCACTTTGGACTCTG | 0 | 8 | 0 | Novel | **Yes(3)** | chr1:143550774-143550873[-] |
| has-miR-B141 | AACTCGTGTTCAAAGCCTTTAG | 0 | 9 | 0 | Novel | No | chr5:171750869-171750890[+] |
| has-miR-B142 | TGTGTGTGCTTGTATATATAT | 0 | 0 | 5 | Novel | No | chr8:10807951-10807971[+] |
| has-miR-B143 | TCATGTTGCTCTGCTGTTG | 3 | 5 | 1 | Novel | No | chr1:229467218-229467236[+] |
| has-miR-B144 | TTAGCCAATTGTCCATCTTTAG | 0 | 6 | 0 | Novel | **Yes(4)** | chr8:125903391-125903493[+] |
| has-miR-B145 | CTGGGTGACAAGAGTGAGACTCCTTCTCT | 3 | 0 | 2 | Novel | No | chr16:30517528-30517556[+] |
| has-miR-B146 | TCAGGAGTAAAGACAGAGTTC | 0 | 7 | 1 | Novel | No | chr11:64056324-64056344[+] |
| has-miR-B147 | CCACTTGGATCTGAAGGCTGCC | 0 | 1 | 7 | Novel | **Yes(9)** | chr17:52323619-52323723[-] |
| has-miR-B148 | CGCGTTGGTGGTATAGT | 8 | 0 | 0 | Novel | No | chr9:134796704-134796720[+] |
| has-miR-B149 | TCTTGGGCCCCACCCCTGGAGAT | 4 | 1 | 3 | Novel | No | chr5:149306518-149306540[+] |
| has-miR-B150 | TAAAACTGCAGTTATTTTTGC | 6 | 0 | 0 | Novel | No | chr13:114028118-114028138[+] |
| has-miR-B151 | GGGTGTTGGGTGCTAGTCGGCACCAGA | 0 | 0 | 6 | Novel | No | chr5:123066211-123066237[+] |
| has-miR-B152 | ATCTCATGCCTGTGCTCCGGA | 4 | 0 | 1 | Novel | No | chr2:3610969-3610989[+] |
| has-miR-B153 | AAGTAATTGCGGGTTTTGCCTTT | 2 | 0 | 3 | Novel | No | chr6:164432277-164432299[+] |
| has-miR-B154 | TTGGCCCTTATCGAAGCTGC | 0 | 0 | 7 | Novel | No | chr1:218469568-218469587[+] |
| has-miR-B155 | CGCGCCTGCAGGAACTGGTAGA | 3 | 0 | 0 | Novel | **Yes(10)** | chr6:1335536-1335637[-] |
| has-miR-B156 | CCAGGCTCTGCAGTGGGAACTG | 0 | 0 | 4 | Novel | No | chr10:6234216-6234237[+] |
| has-miR-B157 | TACATGGATGGAAACCTTCAAGC | 0 | 5 | 1 | Novel | **Yes(7)** | chr4:40198803:40198903[-] |
| has-miR-B158 | CAAAACCGCGATTACTCTTGCA | 2 | 2 | 2 | Novel | No | chr3:166979006-166979027[+] |
| has-miR-B159 | TTGCTGTGATGATGTTCTGAGCTC | 0 | 0 | 4 | Novel | No | chr15:53678389-53678412[+] |
| has-miR-B160 | TTCTCAAGAGGGAGGCAATCAT | 6 | 0 | 0 | Novel | No | chrx:8774327-8774348[+] |
| has-miR-B161 | TGGAAAAAACTGGTGTGTGCTT | 0 | 3 | 0 | Novel | No | chr8:116340430-116340451[+] |
| has-miR-B162 | TGTGGGTTCTGGGTTGGGGTGA | 0 | 0 | 2 | Novel | No | chr19:10800654-10800675[+] |
| has-miR-B163 | TGGTTTTTGTTGTCAGCTGGTTAG | 1 | 0 | 1 | Novel | No | chr6:144764508-144764531[+] |
| has-miR-B164 | TAGCTCTGTCCTTGGGAATCGC | 0 | 0 | 2 | Novel | No | chr16:56327872-56327893[+] |
| has-miR-B165 | CACATATGAAGTGAGCCAGCAC | 0 | 0 | 3 | Novel | No | chr14:52883670-52883691[+] |
| has-miR-B166 | GGTTCTTAGCATAGGAGGTCT | 1 | 1 | 0 | Novel | **Yes(3)** | chr15:57250662-57250762[-] |
| has-miR-B167 | TGCTGTATTGTCAGGTAGTGA | 5 | 0 | 0 | Novel | **Yes(3)** | chr19: 8360167-8360266[-] |
| has-miR-B168 | TGCAGCTGCCCGCCCGGGACGC | 0 | 2 | 0 | Novel | No | chr2:169799256-169799277[+] |
| has-miR-B169 | ACCCTGTCCTCCAGGAGCTCA | 3 | 0 | 2 | Novel | No | chr22:30854336-30854356[+] |
| has-miR-B170 | TTTCTGCTTACATGATCTCTGCA | 3 | 0 | 0 | Novel | No | chr11:29165443-29165465[+] |
| has-miR-B171 | TGGCTGGGTGCAAGGCACG | 4 | 0 | 0 | Novel | No | chr19:54684803-54684821[+] |
| has-miR-B172 | TATGTGACCTCGGATGAATCA | 0 | 4 | 0 | Novel | **Yes(2)** | chr13:95877462- 95877571[+] |
| has-miR-B173 | GAGGGCAGGTGGGGGGC | 3 | 0 | 0 | Novel | No | chr22:8527048-8527064[+] |
| has-miR-B174 | CTGGGTGAGGGCATCTGTGGT | 0 | 3 | 0 | Novel | No | chr1:144295785-144295805[+] |
| has-miR-B175 | TGTGGGACTTCTGGCCTTGACT | 0 | 1 | 3 | Novel | No | chr12:103509593-103509614[+] |
| has-miR-B176 | TGTGATATCGTGCTTCCTGGGA | 0 | 0 | 4 | Novel | No | chr9:3390782-3390803[+] |
| has-miR-B177 | TAGCACAGAATAGTTCAGTTGC | 0 | 2 | 2 | Novel | No | chr5:142286789-142286810[+] |
| has-miR-B178 | CCCTGTGGTGGCGACGAC | 2 | 0 | 0 | Novel | No | chr1:81193591-81193608[+] |
| has-miR-B179 | TTGGGCTGAGGCGGAGGAGCCGCCGC | 2 | 2 | 0 | Novel | No | chr12:22844085-22844110[+] |
| has-miR-B180 | TGGGGAGGGATGGCGGGA | 2 | 0 | 0 | Novel | No | chr11:60892823-60892840[+] |
| has-miR-B181 | TGGAGAGAGAAAAGAGACAGAAG | 0 | 1 | 2 | Novel | No | chr6:170481826-170481848[+] |
| has-miR-B182 | TGGACTGCGCCGCCGGAGCG | 0 | 0 | 2 | Novel | No | chr7:2525936-2525955[+] |
| has-miR-B183 | TCTTCTTCCTTTGCAGAGTTGA | 0 | 3 | 0 | Novel | No | chr2:176512236-176512257[+] |
| has-miR-B184 | TCAAAACTGCAATTACTTTTGC | 3 | 0 | 0 | Novel | No | chr8:70795758-70795779[+] |
| has-miR-B185 | GAGCCAGTGGTGAGACAGTGAG | 0 | 0 | 2 | Novel | No | chr10:74150802-74150823[+] |
| has-miR-B186 | CTAGGTGGGGGGCTTGAAGCC | 0 | 3 | 0 | Novel | No | chr19:3368501-3368521[+] |
| has-miR-B187 | ATCCACAGTGCAGCAAGGGC | 0 | 0 | 2 | Novel | No | chr9:80119566-80119585[+] |
| has-miR-B188 | AGTGTGAGGTTGTCATGCCTGCT | 0 | 3 | 0 | Novel | No | chr2:132907772-132907794[+] |
| has-miR-B189 | TTGCTGGGAAAGGGAGAAGTTC | 0 | 0 | 2 | Novel | No | chr8:144584101-144584122[+] |
