# Supplementary material for: Discovery of Novel Human Breast Cancer MicroRNAs from Deep Sequencing Data by Analysis of Pri-MicroRNA Secondary Structures
Source: PLoS One. 2011 Feb 8;6(2):e16403. doi: 10.1371/journal.pone.0016403 (PMC3035615; doi:10.1371/journal.pone.0016403)
Supplement: Table S3 — A lists of miRNA evaluated by Northern blot. (DOC) [file pone.0016403.s005.doc]

**Table S3. A lists of miRNA evaluated by Northern blot**

| **miRNA**  **candidates** | **Frequency** | **Stem length** | **Northern blot**  **(endogenous RNA)** | **Northern blot**  **(Overexpression )** |
| --- | --- | --- | --- | --- |
| mir-B1 | 1251 | 4 | No | No |
| mir-B2 | 1651 | 18 | No | No |
| mir-B3 | 1007 | 28 | No | No |
| **mir-B4** | **57** | **35** | **No** | **Yes** |
| mir-B5 | 396 | 4 | No | No |
| **mir-B6** | **215** | **45** | **Yes** | **ND*** |
| mir-B7 | 340 | 5 | No | No |
| mir-B8 | 15 | 24 | No | No |
| mir-B9 | 157 | 7 | No | No |
| mir-B10 | 92 | 8 | No | No |
| mir-B12 | 78 | 5 | No | No |
| mir-B14 | 29 | 27 | No | No |
| **mir-B15** | **80** | **56** | **No** | **Yes** |
| mir-B19 | 133 | 28 | No | No |
| **mir-B21** | **88** | **38** | **No** | **No** |
| mir-B23 | 48 | 11 | No | No |
| **mir-B26** | **3** | **35** | **Yes** | **ND*** |
| **mir-B27** | **50** | **31** | **No** | **Yes** |
| **mir-B28** | **8** | **42** | **No** | **Yes** |
| Mir-B30 | 15 | 31 | No | No |

**ND*: not determined by overexpression of precursor**
